# Targeted Lymph Node Immunization with Serotype-Specific Dengue VLP Vaccines Enhances Antibody Avidity and Specificity

**DOI:** 10.3390/vaccines13090941

**Published:** 2025-09-03

**Authors:** Dominik A. Rothen, Alessandro Pardini, Sudip Kumar Dutta, Pascal S. Krenger, Anne-Cathrine Vogt, Romano Josi, Monique Vogel, Paul Engeroff, Mona O. Mohsen, Kaspars Tars, Byron Martina, Martin F. Bachmann

**Affiliations:** 1 Department of BioMedical Research, University of Bern, 3008 Bern, Switzerlandmartin.bachmann@unibe.ch (M.F.B.); 2 Department of Immunology RIA, University Hospital Bern, 3010 Bern, Switzerland; 3 Graduate School of Cellular and Biomedical Sciences, University of Bern, 3012 Bern, Switzerland; 4 Artemis Bioservices, 2629 JD Delft, The Netherlands; 5 Tajarub Research & Development, Doha P.O. Box 12627, Qatar; 6 Latvian Biomedical Research & Study Centre, Ratsupites iela 1, LV 1067 Riga, Latvia; 7 Curacao Biomedical & Health Research Institute, Pater Eeuwensweg 36, Willemstad, Curaçao; 8 Jenner Institute, Nuffield Department of Medicine, University of Oxford, Oxford OX3 7DQ, UK

**Keywords:** dengue, vaccine, lymph node, virus, flaviviruses, virus-like particle, VLP, AP205

## Abstract

Introduction: Dengue virus (DENV) remains a global health threat, with four distinct serotypes (DENV1-4) that complicate vaccine development due to low-affinity, cross-reactive antibodies that increase the risk of antibody-dependent enhancement (ADE). Objective: To address the challenge of inducing strictly serotype-specific immune responses, this study explored the use of targeting individual lymph nodes (LNs) for the creation of simultaneous but independent immune responses as a targeted approach to reduce cross-reactivity and improve vaccine specificity. Methods: In the initial experiments, targeting individual LN successfully induced specific germinal centers (GCs) for different antigens in distinct LNs, highlighting its potential to enhance immune specificity. This approach was further tested using two virus-like particle (VLP)-based vaccines based on AP205 for DENV1 and DENV4, selected due to their genetic divergence and to probe the potential to minimize cross-reactive immune responses. In this setup, AP205-DV1 and AP205-DV4 were administered in targeted separate LNs, and the specificity of the immune response was compared to subcutaneous administration of a mixture of both vaccines. Results: Our data show that targeting distinct LNs elicited antibodies with significantly higher avidity, which is a critical factor in determining the neutralizing capacity of the immune response. Avidity measurements confirmed that this segregation approach results in a more refined selection of high-affinity B cells. Neutralization experiments demonstrated that targeting distinct LNs with individual vaccines induced a more potent and serotype-specific neutralizing response, compared to the injection of a vaccine mixture. Conclusions: These findings suggest that targeting individual LNs could be a promising method for enhancing both the specificity and potency of immune responses, particularly for flaviviruses. Targeting distinct LNs by direct administration of individual vaccines into distinct watersheds rather than individual lymph nodes will offer the opportunity to facilitate the approach in humans.

## 1. Introduction

Dengue fever, caused by the dengue virus (DENV), remains a significant global health challenge, particularly in tropical and subtropical regions [1]. Despite extensive efforts, effective and universally applicable vaccines have remained elusive, highlighting the need for innovative approaches to vaccination strategies. Many viral species exhibit high genetic variability, which allows them to evade virus-neutralizing antibody responses [2]. A more evolved strategy for immune evasion, as is the case for dengue, is the creation of distinct serotypes, which resulted in the emergence of four dengue serotypes [3,4]. This serotype diversity complicates the induction of protective immune responses, as cross-reactive, non-neutralizing antibodies can lead to antibody-dependent enhancement (ADE) [5]. ADE facilitates viral entry into host cells via Fcγ receptors, increasing replication and potentially causing more severe disease [6]. This phenomenon poses challenges for dengue vaccine development, requiring a vaccine to elicit a protective response against all four serotypes with minimal induction of cross-reactive non-neutralizing antibodies. Hence, in the case of dengue, the goal is to simultaneously induce highly specific antibodies singularly recognizing one of the four existing serotypes with insignificant cross-reactivity. Previous antigenic exposure can influence the induction of specific antibodies for multiple dengue serotypes [7]. Upon infection with a new dengue serotype, the immune system preferentially activates memory B cells generated from a previous infection. These memory B cells produce antibodies specific to the initial serotype, thereby restricting the production of new serotype-specific antibodies. This phenomenon, known as original antigenic sin or molecular imprinting, complicates the development of effective vaccines targeting all four dengue serotypes. Furthermore, simultaneous immunization against all serotypes tends to favor the induction of cross-reactive B cells, as these cells are more likely to recognize and respond to antigens from all four serotypes. Consequently, both sequential immunization and immunization with a cocktail of serotype-specific vaccines promote the production of antibodies targeting cross-reactive epitopes [8,9]. In a previous study, we tried to overcome this challenge by developing virus-like particles (VLP)-based DENV vaccines that display the Domain III (EDIII) of the envelope protein rather than the entire envelope protein, since EDIII has fewer cross-reactive epitopes and is highly serotype-specific; the vaccine candidates indeed induced a potent and specific neutralizing antibody response [10,11,12]. VLPs are multi-protein structures that mimic many characteristics of viruses and are used in vaccines for Hepatitis B, Hepatitis E, human papilloma virus (HPV), and malaria [13,14]. They can display complex antigens in native form in a repetitive manner upon directed chemical coupling or genetic fusion and lack replication-competent genetic material, making them a safe platform for immunocompromised individuals [15,16,17]. Their nanoparticle size of 20–200 nm allows them to efficiently reach draining lymph nodes (dLNs), inducing strong innate and adaptive immune responses across various species [15,17,18]. To accommodate the larger EDIII domain, we genetically fused or chemically coupled the EDIII of the 4 DENV serotypes to AP205-VLPs with two monomers fused, resulting in 90 rather than 180 N/C-termini, presenting a sterically optimized VLP, as described in our previous study [19,20].

In a previous study, we demonstrated that simultaneous vaccination with a tetravalent mixture of serotype-specific VLP vaccines could induce a potent humoral immune response able to efficiently neutralize all four DENV serotypes without enhancing DENV-2 replication [12]. However, studies on the induction of cross-reactive antibodies by the vaccines revealed considerable cross-reactivity. Since all four vaccines are presented to the immune system and are available to follicular dendritic cells (FDCs) in GCs, we still expect a preferential induction of cross-reactive B cells.

This study presents a novel approach to improve serotype-specific responses to anti-EDIII vaccines by inducing specially segregated immune responses by targeting individual LNs with individual serotype-specific vaccine candidates. As a proof-of-concept, we immunized with two dengue serotypes, DENV-1 and DENV-4. These represent the most genetically and antigenically distant serotypes, making them suitable to investigate the mechanisms underlying the immune response induced by lymph node-targeted vaccination. Since LNs function as independent entities, particularly those in separate areas [21], it is possible to simultaneously induce a distinct, independent immune response in individual LN regions. This approach allows for the concurrent induction of highly serotype-specific B-cell responses within the same host. Therefore, targeting individual LNs presents a promising strategy for eliciting uniquely serotype-specific immune responses, making it a potentially effective approach for dengue vaccination.

## 2. Materials and Methods

### 2.1. Mice

Wild-type C57BL/6JOlaHsd mice and BALB/cOlaHsd, sourced from Envigo, were used for the in vivo experiments, involving female mice aged between 8 and 12 weeks. All animal procedures adhered to the Swiss Animal Act (455.109.1—September 2008, 5th revision) and the protocols were approved by the Swiss Federal Veterinary Office.

### 2.2. Generation of Vaccines

Design and production of the vaccines used in this study are described in Rothen et al. (2024) [12].

### 2.3. LNs Targeted Vaccination

Wild-type C57Bl/6JOlaHsd and BALB/cOlaHsd mice (8–12 weeks, Envigo) were used to perform this experiment. The mice were anaesthetized with 50 µL/25 g mice of anaesthesia mix s.c. (Dormitor 1 mg/mL, Dormicum 5 mg/mL, Fentanyl 0.05 mg/mL). After waiting 7–10 min until the mice were completely anaesthetized, Ophthalmic ointment (e.g., Paralube^®^ or Lacrilube^®^) was applied to the mice’s eyes to avoid drying out of the cornea. To ensure that the mouse was sufficiently anaesthetized, we pinched its foot or toe with a finger. If no reflexes were detected, we proceeded with the procedure. If reflexes were detected, we waited an extra 5 min and checked the mouse again. The mouse was placed on its back, the area for surgery (inguinal region) was shaved (with cream or trimmer), and disinfection was carried out with Betadine. The hip joint was bent to approximately a 90° angle, and a small incision of <5 mm through the skin was made using sterile curved micro-dissecting forceps and a surgical scissor. The incision was widened using the tip of the surgical scissors, which tore the skin to about 10 mm. The inguinal lymph node was localized and immobilized using the curved forceps and the surgical scissors. The inguinal lymph node appeared greyish within the surrounding fat tissue. Using an insulin syringe (0.5 mL, 30 G), 10 µL of the vaccine was directly injected into the inguinal lymph node. If the lymph node was getting swollen, the injection was assumed successful. The incision was sealed using sterile 9 mm wound clips inserted into a 9 mm Auto-clip applier. Clips were removed after 10–12 days. The mice were then boosted with the same method.

### 2.4. Vaccination Regimen

For the first experiment, wild-type BALB/cOlaHsd mice (8–12 weeks old, Envigo) were utilized. In the LN-targeted group, each mouse received a lymph node-targeted injection of 10 µg of AP205 into the inguinal lymph node in the right leg (right LN) and 10 µg of QB into the left leg’s lymph node. In the s.c. group, a mixture of 10 µg AP205 and 10 µg QB was injected into the right lymph node. All injections were administered in a total volume of 20 µL without adjuvants. Lymph nodes were harvested on day 9 for analysis. In the second experiment, wild-type C57BL/6JOlaHsd mice (8–12 weeks old, Envigo, Amsterdam, The Netherlands) were used. In the LN-targeted group, 10 µg of AP205-DV1 was injected into the left lymph node, and 10 µg of AP205-DV4 was administered into the right lymph node. In the s.c. group, a mixture of 10 µg AP205-DV1 and 10 µg AP205-DV4 was injected subcutaneously. Mice were boosted in the same manner on day 28 and terminally bled on day 49. Weekly tail bleeds were performed to collect serum, which was separated in Microtainer tubes for further analysis.

### 2.5. Immunohistochemistry

To conduct immunofluorescent staining, LNs were initially embedded in OCT media (Sakura, Tokyo, Japan, cat.4583) and frozen on dry ice. Samples were stored at −20 °C for up to one week or longer at −80 °C, then moved to −20 °C for at least a day before sectioning. Tissue sections, 8 µm thick, were cut using a cryostat (Thermo Fisher Scientific, Waltham, MA, USA) and placed onto Superfrost Plus glass slides (Epredia, Campus Dr, Kalamazoo, MI, USA), where they were allowed to dry overnight at room temperature before staining. The sections were fixed by immersing them in 100% cold acetone for 10 min at 4 °C. Following fixation, they were air-dried, and a SuperHT PAP Pen (biotium, Landing Parkway, Fremont, CA, USA) was used to encircle the sections. Sections were air-dried for 10 min, then rehydrated by incubating in PBS for 5 min. To block nonspecific binding, 100 µL of blocking buffer (1% BSA and 1% normal mouse serum in PBS) was applied to each section and incubated for 20 min at room temperature. The excess blocking buffer was removed by gently blotting the edge of the slide on paper towels. Then, 90 µL of primary antibody (bio-PNA diluted 1:500 in PBS with 1% NMS and 0.1% BSA) was applied to each section, and the slides were incubated for 60 min in the dark when fluorescently labelled antibodies were used. After incubation, the slides were washed three times in PBS for 5 min with gentle agitation. The secondary antibody mixture (QB-Alexa-488 or AP205-Alexa-488 (self-made), Streptavidin-550 (Mabtech, Nacka Strand, Sweden, cat. 3310-11-1000), and B220-Alexa-647 (BioLegend, San Diego, CA, USA, cat. 103224), diluted in PBS with 1% NMS and 0.1% BSA) was then applied to the sections, which were incubated for 45 min in the dark. The same washing procedure was repeated. After removing excess liquid, a drop of Fluoromount-G mounting medium (Thermo Fisher Scientific, Waltham, MA, USA, cat. 00-4958-02) was added, and a coverslip was carefully placed over the sections to avoid bubbles. The coverslip edges were sealed with nail varnish, and the slides were stored in the dark at 4 °C. Images were captured within two days to preserve fluorescence with the Zeiss Axio Imager A2 microscope using the AxioVision software (Zeiss, Oberkochen, Germany, Version 4.8.2) and analysed with ImageJ (Version 1.54p).

### 2.6. Enzyme-Linked Immunosorbent Assay (ELISA)

To quantify total IgG titers, Corning™ 96-Well Half-Area Plates (Thermo Fisher Scientific, Waltham, MA, USA) were coated with 50 µL/well of DENV1 or DENV4 envelope protein domain III (1 µg/mL in PBS) and incubated overnight at 4 °C on a shaker. The following day, plates were washed, then blocked with 100 µL/well of PBS containing 0.15% casein for 2 h at room temperature with shaking.

Serum samples were added at an initial 1:20 dilution, followed by 1:3 serial dilutions across the plate. Plates were incubated for 2 h at room temperature with shaking, then washed again with PBS + 0.01% Tween using an ELISA washer (BioTek, Winooski, VT, USA, 405 TS). A secondary HRP-conjugated anti-mouse IgG (Jackson ImmunoResearch, West Grove, PA, USA, Cat. No. C840T69) was added at a 1:1000 dilution and incubated for 1 h. After a final wash, plates were developed, and absorbance was measured at 450 nm using a BioTek microplate reader. The OD50 values were calculated as the reciprocal of the 50% dilution relative to the maximum OD450 value.

### 2.7. Avidity (ELISA)

To assess the avidity of IgG antibodies, two sets of plates were prepared. Both sets were coated with 1 µg/mL of DENV envelope protein domain III. After serum incubation, one set was washed three times for 5 min with 50 µL/well of 7 M urea in PBS + 0.05% Tween-20, while the other set was washed with the same amount of PBS + 0.05% Tween-20. Between these washing steps, all plates were washed using an ELISA washer with PBS + 0.01% Tween-20. The remaining steps followed the standard ELISA protocol as previously described. The avidity index was calculated as the ratio of the OD450 signal from urea-washed wells to the signal from control-washed wells, expressed as a percentage.

### 2.8. FluoroSpot

On day 1, dengue virus EDIII (DV) proteins were diluted in sterile PBS to a final concentration of 50 µg/mL, with 100 µL of the solution added per well of a FluoroSpot plate (Mabtech, Nacka Strand, Sweden, Cat no. 3654-FL). The membrane of the FluoroSpot plate was pre-wet with 35% ethanol (EtOH), 15 µL/well, for no longer than 1 min to ensure membrane integrity. After pre-wetting, the plate was washed 5 times with sterile water (200 µL/well). Then, the DV protein solution was added (100 µL/well) and incubated overnight at 4 °C. On Day 2, plates were washed five times with PBS (200 µL/well) and blocked with 200 µL/well of RPMI medium supplemented with 10% FBS for at least 30 min at room temperature. On the same day, mice were euthanized for terminal blood collection, and lymph nodes were harvested into 2 mL of RPMI containing 2% FCS and antibiotics. A single-cell suspension was created by passing the lymph node through a 70 µm cell strainer, and the resulting suspension was collected in a Falcon tube. Additional RPMI medium was used to wash the petri dish, and the wash was added to the tube. The cells were centrifuged for 8 min at 4 °C and 300× *g*. The supernatant was discarded, and the cell pellet was resuspended in 1 mL RPMI with 2% FCS and antibiotics. Cells were diluted to 5 × 10^6^ cells/mL for use in the assay. Before cell incubation, the medium was removed from the pre-coated wells, and 500,000 cells were seeded per well. The plates were incubated at 37 °C with 5% CO_2_ for 20 h, protected from light. After incubation on the next day, the cells were removed, and the plate was washed 5 times with PBS (200 µL/well). The primary antibody, goat anti-mouse IgG-biotin (diluted 1:1000 in PBS-0.1% BSA) (Southern Biotech, Birmingham, AL, USA, Cat no. 1030-08), was added to the wells (100 µL/well) and incubated for 2 h at room temperature. The plate was then washed 5 times with PBS, followed by the addition of Streptavidin-550 (diluted 1:200 in PBS-0.1% BSA, 100 µL/well) (Mabtech, Nacka Strand, Sweden, Cat no. 3310-11-1000), and incubated for 1 h at room temperature. After a final wash, 50 µL/well of fluorescence enhancer (Mabtech, Nacka Strand, Sweden, Cat. No. 3641) was added for 5–15 min at room temperature. The enhancer was discarded by flicking the plate (without washing or blotting), and plates were allowed to dry in the dark at room temperature. Plates were stored protected from light and read at 550 nm using a FluoroSpot reader (Mabtech, Nacka Strand, Sweden, IRIS).

### 2.9. Cell Culture

The C6/36 mosquito cell line (CLR-1660, American Type Culture Collection, ATCC) was maintained in Eagle’s Minimum Essential Medium (EMEM) (Lonza, Basel, Switzerland) supplemented with 10% heat-inactivated fetal bovine serum (HI-FBS, Lonza Benelux BV, Breda, The Netherlands), 0.75% sodium bicarbonate (NaHCO_3_, Lonza), 10 mM HEPES buffer (Lonza), and 1% penicillin–streptomycin (Pen-Strep, Lonza). The cells were cultured at 28 °C in a non-CO_2_ incubator. Vero cells (ATCC^®^ CCL-81™, Manassas, VA, USA) were grown in Dulbecco’s modified Eagle medium (DMEM) supplemented with 10% HI-FBS (Lonza Benelux BV, Breda, The Netherlands), 0.75% NaHCO_3_, 10 mM HEPES buffer (Lonza), and 1% Pen-Strep (Lonza) at 37 °C in a humidified incubator with 5% CO_2_. SC cells (CLR-3622, ATCC) were cultured in RPMI 1640 medium containing 50 µM beta-mercaptoethanol, 100 U/mL penicillin, 100 µg/mL streptomycin (both from Lonza), and 10% HI-FBS (Gibco/ThermoFisher, Paisley, UK) at 37 °C in a humidified incubator with 5% CO_2_. All three cell lines were regularly tested for mycoplasma contamination using an in-house developed RT-PCR assay [22].

### 2.10. Virus

Dengue virus 1 (VR1856, Hawaii) was initially propagated in C6/36 cells. Following viral amplification, the supernatant containing the virus was used to infect Vero cells at a multiplicity of infection (MOI) of 0.01. After 72 h of incubation, the viruses were harvested. Dengue serotype 4 (VR-1490, H241, tissue culture-adapted) was directly amplified in Vero cells at an MOI of 0.01, and the virus was collected once a cytopathic effect was observed. The virus stocks were clarified by centrifugation and stored at −80 °C for future use. To determine viral titers, 10-fold serial dilutions of DENV1 and DENV4 stocks were incubated on Vero cells for 4 days at 37 °C with 5% CO_2_. Infected cells were fixed with 2.5% formalin and permeabilized using 0.1% Triton X-100 in 70% ethanol. Viral infection was detected using a rabbit anti-flavivirus group antigen monoclonal antibody (Absolute Antibody, Oxford, UK), followed by a goat anti-rabbit IgG Alexa Fluor Plus 488 conjugate (Invitrogen, Waltham, MA, USA). Nuclei were counterstained with DAPI (4′,6-diamidino-2-phenylindole dihydrochloride). Plates were scanned at 4× magnification using a Cytation 1 V Imaging Reader (BioTek, Winooski, VT, USA), and image analysis was performed with Gen5 software v2.1 (BioTek, Winooski, VT, USA). The virus titers were then calculated using the Karber formula [23].

### 2.11. Focus Reduction Neutralization Test (FRNT)

To evaluate the neutralizing capacity of sera from vaccinated mice, a focus reduction neutralization test (FRNT) was performed. Human convalescent serum 001 against dengue virus (NR-50226) served as a positive control, while serum from naïve C57BL/6JOlaHsd mice was used as a negative control. One day prior to infection, Vero cells were seeded in 96-well plates at a density of 2 × 10^4^ cells/well in complete medium to form a confluent monolayer. To inactivate complement, the serum from vaccinated mice was heat-inactivated at 56 °C for 30 min and then serially diluted 1:2 in infection media (DMEM with 0.75% NaHCO_3_, 10 mM HEPES buffer, 1% Pen-Strep, and 1% HI-FBS), with dilutions ranging from 1:20 to 1:160. Each dilution was mixed with 500 TCID_50_ of the respective dengue virus serotype and incubated at 37 °C for 1 h to allow antibody–virus interaction. Following incubation, the virus–serum mixtures were transferred to the pre-seeded Vero cell plates and incubated for 1 h at 37 °C in a 5% CO_2_ atmosphere. The monolayer was then washed once with PBS, and fresh infection medium was added. Plates were incubated for an additional 48 h under the same conditions. An in-house immunofluorescence assay was used to determine the percentage of infected cells. The FRNT50 titer of each sample was calculated by comparing the number of infected cells to the virus control, and each sample was tested in duplicate.

### 2.12. Statistical Analysis

Data were analyzed and presented as mean ± SEM using unpaired Student’s *t*-test or one-way ANOVA with Tukey correction for multiple comparisons as mentioned in the figure legend using GraphPad PRISM (GraphPad Software Inc., Version 10.3.1). The value of *p* < 0.05 was considered statistically significant (* *p* < 0.05, ** *p* < 0.01, *** *p* < 0.001, **** *p* < 0.0001).

## 3. Results

### 3.1. Targeted Immunization of Individual LNs Induces Spatially Segregated, Individual Immune Responses Against AP205- and QB-VLPs

**Figure 1:**
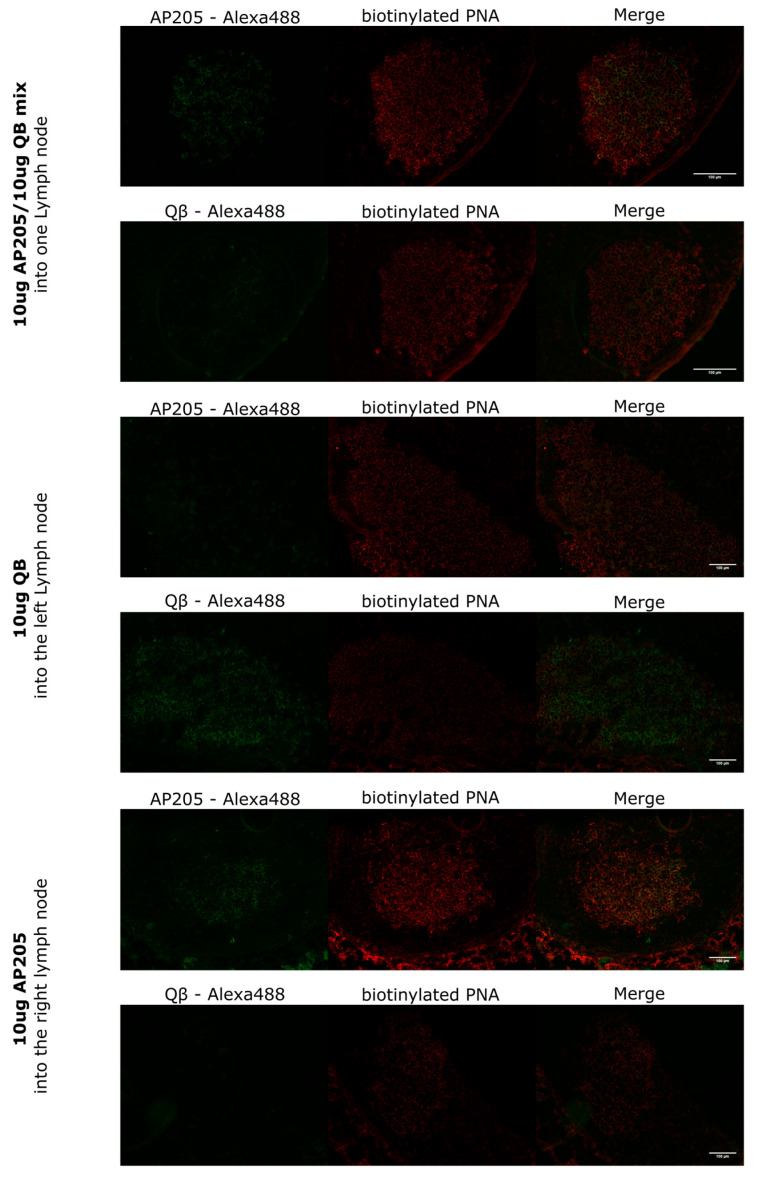
Assessment of AP205- and QB-VLPs response in GCs in the LNs. GCs are visualized in red by PNA-binding GC B cells. QB- or AP205-binding cells are shown in green, stained with QB-Alexa488 or AP205-Alexa488. Eight-week-old Balb/c mice were immunized with 10 µg AP205- and 10 µg QB-VLPs as a mix into the same individual lymph node (shown in the (**first column**)) or 10 µg QB into the left lymph node (**second column**) and 10 µg AP205 into the right lymph node (**third column**). Lymph nodes were taken 9 days after the prime. Original magnification 20× for the first column and 10× for the second and third columns. The scale bar in white is set at 100 µm. The experiment was performed twice.

To investigate localized germinal center responses in individual LNs, 8-week-old BALB/c mice were immunized with AP205 and QB VLPs, either as a mixture into a single lymph node or as separate administrations into distinct lymph nodes, allowing for a direct comparison of the germinal center reactions elicited by targeted immunization. Given that lymph nodes function as independent entities, especially when located in separate regions [21], this approach aims to target the induction of distinct germinal center responses in each lymph node following immunization. Figure 1 illustrates the GC responses to AP205-VLPs and QB-VLPs following immunization. The mixed VLPs administered into a single LN showed GCs that display a double-positive reaction, characterized by a strong binding by B cells of AP205-VLPs and QB-VLPs in the same GC. For targeted immunization into separate LNs, we chose the left and right inguinal LNs, which are located in separate watersheds in mice. This approach will ensure successful separate immunization. In contrast, when QB-VLPs were injected into the left inguinal LN, the resulting GC was specific to QB-VLPs, showing no AP205-VLPs signal. Conversely, the right inguinal LN, which received AP205-VLPs, exhibited a GC response exclusively specific to AP205-VLPs. Confirming that each LN developed a distinct antigen-specific response based on the localized immunization.

### 3.2. Lymph Node Targeted Vaccination of AP205-DV1 and AP205-DV4 Separately into One Lymph Node Elicits Abs of Higher Avidity Compared to Subcutaneous Administration of Vaccine Mixture

**Figure 2:**
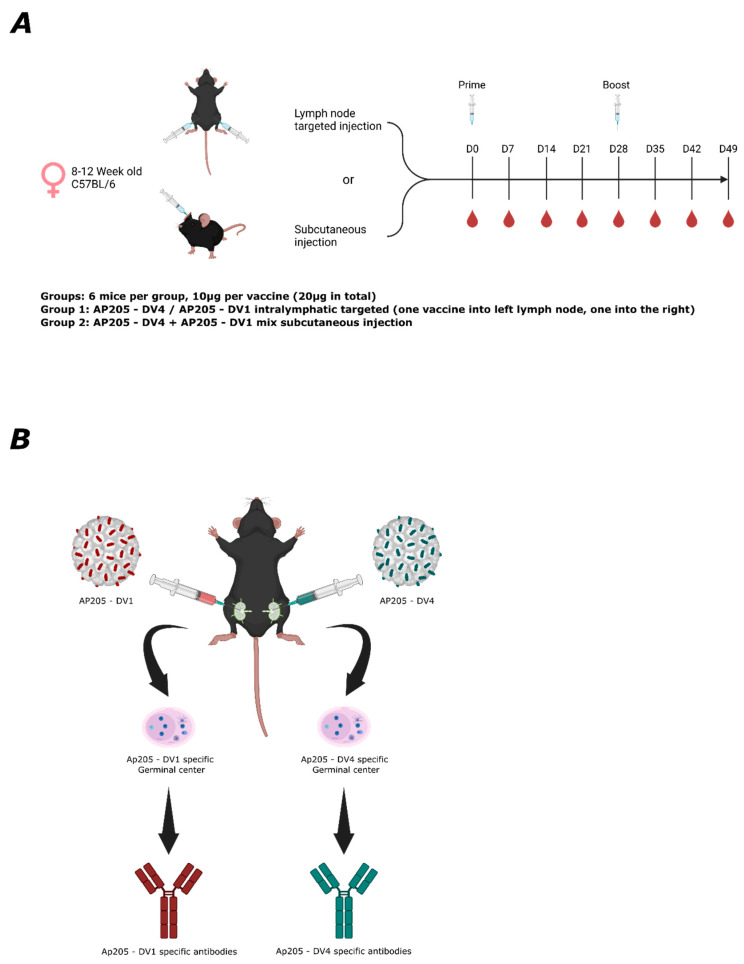
Vaccination regimen and experimental design for LN targeted and s.c. vaccination: (**A**) Vaccination overview. C57BL/6 Mice were either LN target vaccinated or s.c. immunized, as shown in the schematic, at day 0 with 20 µg of total vaccine (10 µg per LN) and boosted on day 28. Blood samples were taken weekly, and the experiment terminated on day 49. (**B**) Experimental design of lymph node-targeted injection. AP205-DV1 and AP205-DV4 were injected into the left LN and the right LN to induce dengue-specific GCs, producing specific antibodies. Figures created with https://www.biorender.com/.

In the next step, we assessed the humoral immune response elicited by our generated dengue vaccines, genetically incorporating the EDIII domain [12]. Specifically, we focused on measuring IgG-specific immune response following segregated vaccination in separate LNs or after s.c. administration of the vaccine mixture. Measuring antibody avidity after vaccination is essential to assess the overall strength of binding between an antibody and the antigen vaccinated against, which correlates with neutralization efficiency. Thus, we assessed the avidity in our two regimens. Figure 2A depicts the vaccination regimen used in the experiment. C57BL/6 mice were either LN targeted or subcutaneously injected on day 0 as a prime and then boosted on day 28 using the same approach. In LN targeted vaccination, 10 µg of AP205-DV1 was injected into the inguinal LN of the left limb, while 10 µg of AP205-DV4 was injected into the inguinal LN of the right limb. In the s.c. vaccination group, 10 µg of AP205-DV1 was mixed with 10 µg of AP205-DV4 and administered as a single s.c. injection. Figure 2B illustrates the experimental design for LN-targeted vaccination, where individual vaccinations aim to induce GC that produce individual vaccine-specific antibodies. The DV1- and DV4-specific IgG responses were assessed using ELISA.

**Figure 3:**
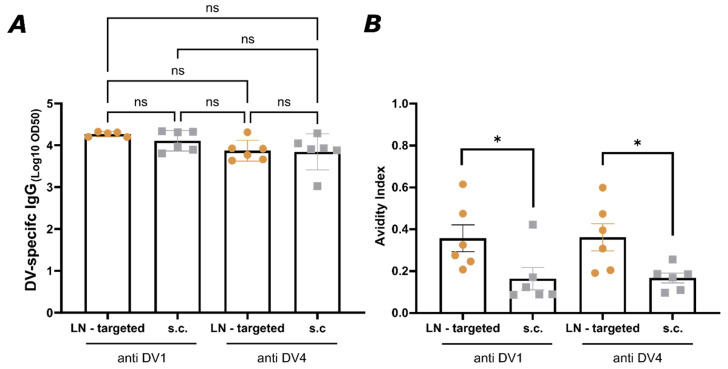
Lymph node targeted vaccination of AP205-DV1 and AP205-DV4 separately into one lymph node elicits antibodies of higher avidity compared to subcutaneous injection of the vaccines as a mixture: (**A**) DV1- and DV4-specific IgG titer. LN—the targeted group (golden) was vaccinated with 10 µg AP205-DV1 and 10 µg AP205-DV4 into two separate lymph nodes. S.c. group (silver) was vaccinated with 20 µg AP205-DV1/AP205-DV4 as a mixture. (**B**) Avidity index of DV1- and DV4-specific IgG titer. Sera was used from day 49. Titer was measured by ELISA, with the LOG10 OD50 shown. Statistical analysis (mean ± SEM) using Student’s *t*-test. Vaccine groups *n* = 6. The experiment was performed twice (*n* = 2). The value of *p* < 0.05 was considered statistically significant (* *p* < 0.05). ns, not significant.

In Figure 3A, IgG levels from serum collected on day 49 were measured, and no significant difference in IgG production was observed between the two groups for both DV1 and DV4. Indeed, both vaccination strategies induced a robust and potent specific IgG response if assessed by conventional ELISA. Next, the avidity of the induced IgG antibodies was evaluated (Figure 3B). The avidity index, representing the percentage of high-avidity antibodies, was determined by an avidity ELISA. Strikingly, LN-targeted mice exhibited much higher average antibody avidity (>2-fold) against the EDIII domain of both DENV1 and DENV4 than mice immunized s.c. with the mixture.

### 3.3. Lymph Node Targeted Vaccination Induces Specific Plasma Cells

**Figure 4:**
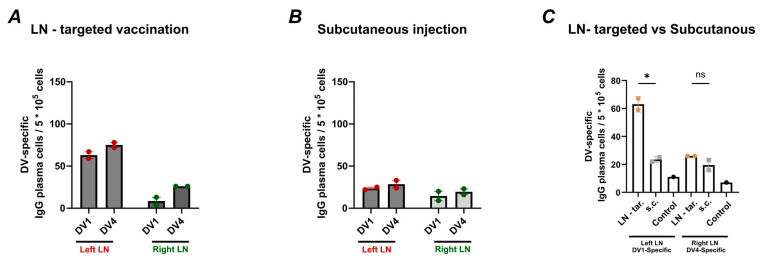
LN-targeted vaccination of AP205-DV1 and AP205-DV4 induces more DV1- and DV4-specific plasma cells in the corresponding lymph nodes compared to subcutaneous vaccination: (**A**,**B**) Number of DV-specific IgG-secreting cells per 500,000 seeded cells in the lymph node of (**A**) L.N-tar. administered and (**B**) s.c. administered. (**C**) Number of DV1- and DV4-specific IgG-secreting cells per 500,000 seeded cells in the left lymph node and the right lymph node. Statistical analysis (mean ± SEM) using Student’s *t*-test. Vaccine group *n* = 6. Control group *n* = 6. The experiment was performed twice (*n* = 2). The value of *p* < 0.05 was considered statistically significant (* *p* < 0.05). ns, not significant.

Induction of specific plasma cells against DENV1 and DENV4 was assessed next. To this end, a FluoroSpot assay was performed to quantify the number of DV1- and DV4-specific plasma cells in the LNs (Figure 2B) of mice vaccinated either lymph node targeted or s.c. Plasma cells play a critical role in short- and long-term immunity by secreting specific antibodies, and their presence in the LNs provides insights into the localized immune response. This experiment aimed to determine whether lymph node targeted vaccination with AP205-DV1 and AP205-DV4 could induce a stronger and more specific response by increasing numbers of DV1- and DV4-specific plasma cells, respectively, compared to s.c. vaccination. Figure 4A–C presents the results found for DV-specific IgG-secreting plasma cells, measured per 500,000 seeded cells. LN-targeted mice exhibited a prominent response, particularly in the left LN where AP205-DV1 was injected, showing a marked increase in DV1-specific plasma cells. In contrast, s.c.-immunized mice demonstrated a more evenly distributed plasma cell response across the two LNs, consistent with the injection of a vaccine mixture. Figure 4C compare the DV1-specific plasma cells in the left LN and DV4-specific plasma cells in the right LN between the two groups.

### 3.4. Targeting Individual LNs Induces an Enhanced DENV-Neutralizing Immune Response Compared to Subcutaneous Injection

**Figure 5:**
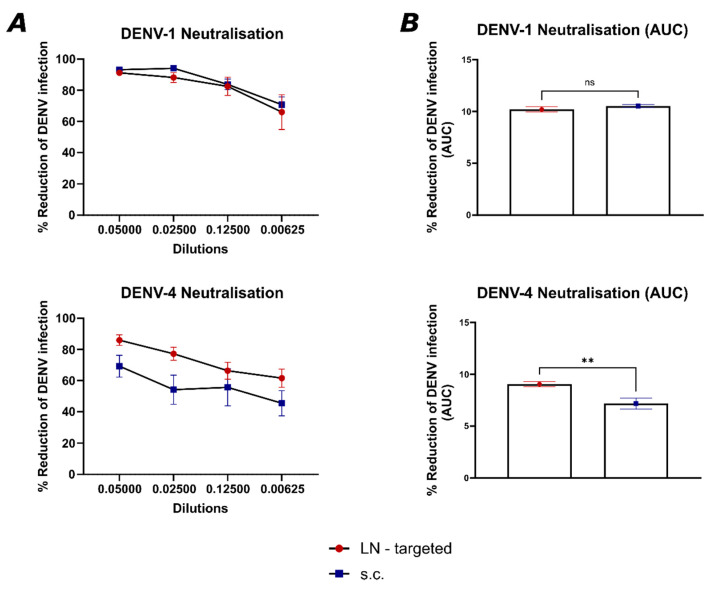
LN-targeted vaccination induces an enhanced DENV-neutralizing immune response compared to subcutaneous injection: (**A**) DENV1 and 4 infection reduction by D49 sera from LN-targeted and s.c. immunized mice at different dilutions. (**B**) Corresponding area under the curve from the reduction in DENV infection graphs DENV 1 (*p* = 0.2924) and 4 (*p* = 0.0085) neutralization. Statistical analysis (mean ± SEM) using Student’s t-test. Vaccine group *n* = 6. Control group *n* = 6. The experiment was performed twice (*n* = 2). The value of *p* < 0.05 was considered statistically significant (** *p* ≤ 0.01). ns, not significant.

To further characterize the immune response elicited by the two vaccination strategies, neutralization assays were performed to evaluate the ability of the sera from both groups to neutralize DENV1 and DENV4. Neutralization is a commonly used measurement of vaccine efficacy, as it directly reflects the functional capacity of antibodies to prevent viral infection [24,25]. Figure 5 compares the neutralization efficiency of sera from mice vaccinated into individual LNs or subcutaneously against DENV1 and DENV4 across multiple dilutions (Figure 5A). The data demonstrate that targeting individual LNs induces a more consistent and potent neutralizing response (*p* = 0.0085) compared to s.c. immunized mice, in particular for DENV4. For DENV1, we observed a similar neutralization efficacy (*p* = 0.2924) between the LN-targeted and s.c. immunized groups among all the dilutions. Especially against DENV4, we can observe a more consistent neutralization induced by targeting individual LNs in contrast to s.c. immunization (Figure 5B).

## 4. Discussion

**Figure 6:**
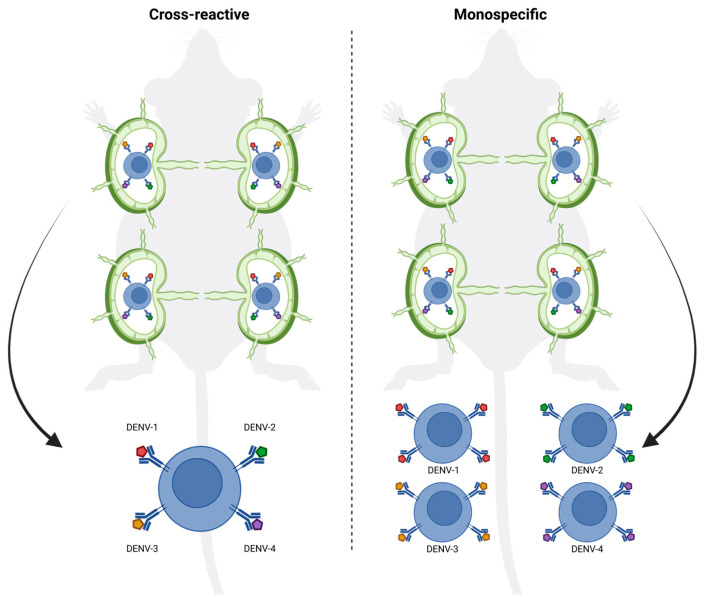
Lymph node-targeted vaccination enhances the specificity of germinal center responses. Schematic representation of conventional vaccination (**left**) versus lymph node (LN)-targeted vaccination (**right**) in mice. Targeted delivery of antigens to distinct lymph nodes focuses germinal center formation, promotes affinity maturation toward a single neutralizing specificity, and reduces cross-reactive B cell selection, leading to higher-avidity antibody responses. Figure created with https://www.biorender.com/.

DENV is one of the most threatening and widespread mosquito-borne viral infections, affecting millions of people each year, particularly in tropical and subtropical regions. The disease presents a significant global health burden, with nearly half the world’s population at risk of infection [26]. A major challenge in dengue vaccine development is the presence of four distinct serotypes [27,28], where immunity to one does not protect against the others and can even increase the risk of severe disease through ADE [25]. Hence, a key goal in dengue vaccine development is to induce a highly specific and potent immune response that effectively neutralizes each serotype while minimizing cross-reactivity that could lead to ADE [29]. Achieving this requires innovative strategies that promote the generation of serotype-specific antibodies with high avidity and neutralizing capacity. Here, we show that targeting individual LNs with individual vaccines offers a promising approach to enhance the precision of the immune response, allowing for the induction of more focused germinal centers and higher-quality antibodies specific to each tested dengue serotype. By ensuring that antigens are delivered directly to distinct LNs, an LN-targeted injection can enhance the specificity of germinal center formation, minimizing cross-reactive antibody responses due to the absence of selection of B cells that react to both serotypes in the same GC (Figure 6) [30].

Indeed, targeting individual LNs by vaccination offered a significant advantage by focusing the immune response on the specific antigen, or in this case, serotype-specific EDIII, presented in each LN, causing higher avidity and better neutralizing antibody responses. This suggests that the LN targeted route allows for more specific germinal center reactions, driving affinity maturation more efficiently to a single neutralizing specificity. The focused antigen presentation on follicular dendritic cells in individual LNs likely plays a role in enhancing this process by ensuring that antigen-specific B cells are selected for affinity maturation without interference from cross-reactive responses (Figure 6). This is particularly important in viruses with high cross-reactivity between serotypes like dengue, where affinity maturation could otherwise be hindered by the presence of broadly reactive but low-affinity B cells stimulated by multiple EDIII domains simultaneously. Moreover, targeting individual LNs by vaccination may help to overcome the phenomenon of original antigenic sin or molecular imprinting, where the immune system preferentially recalls memory B cells from previous infections rather than generating new, serotype-specific responses. By directing antigens to separate lymph nodes, vaccination could encourage the generation of new B cell clones that are specific to the presented serotype rather than activating cross-reactive memory B cells from a previous infection. This could lead to the production of more serotype-specific antibodies and reduce the interference caused by prior immunity. Further testing in animal challenge models will be necessary to evaluate whether this approach can protect against all four dengue serotypes. In addition, dedicated studies will be required to formally demonstrate that this strategy does not promote antibody-dependent enhancement. A further limitation of the present work is that we focused on proof-of-concept immunogenicity against DENV-1 and DENV-4 rather than assessing the durability of the induced immune responses in the context of other serotypes or different mixtures of serotypes.

Conclusion: Targeting individual lymph nodes for dengue vaccination enhances germinal center specificity and affinity maturation, leading to higher-avidity, serotype-specific neutralizing antibodies. Injecting individual LNs in the field for vaccination against DENV is an obvious practical obstacle. However, using adjuvants forming local depots or harnessing natural watersheds in the immune system may be promising and straightforward solutions for the realistic development of such a vaccination strategy in humans. Furthermore, this strategy may not only be attractive for DENV but also other flaviviruses and viruses in general that are thought to cause enhanced disease due to cross-reactive antibodies.

## Data Availability

The original contributions presented in this study are included in the article. Further inquiries can be directed to the corresponding author(s).
